# Robotic-Assisted Surgery in Skull-Base Procedures: Advances, Applications, and Emerging Innovations

**DOI:** 10.1055/s-0046-1818631

**Published:** 2026-04-30

**Authors:** Morad Faoury, Parag Patel

**Affiliations:** 1 Department of Otorhinolaryngology – Head and Neck Surgery, St George's University Hospitals NHS Foundation Trust, London, United Kingdom

**Keywords:** robot-enhanced procedures, technology, skull-base surgery, minimally invasive surgical procedures, artificial intelligence

## Abstract

**Introduction:**

Robotic-assisted surgery (RAS) is increasingly used in skull-base procedures due to its enhanced visualization, precision, and ergonomics, particularly in anatomically-constrained regions. The present narrative review explores the current role and emerging frontiers of RAS in anterior and lateral skull-base surgeries within otolaryngology and neurosurgery.

**Methods:**

We conducted a comprehensive narrative review of studies published throughout the past 2 decades on the PubMed, Scopus, and Web of Science databases using keywords such as *robotic skull-base surgery* , *transoral robotic surgery* , and *robotics in otolaryngology* . We included studies detailing robotic interventions in anterior and lateral skull-base pathology.

**Results:**

Robotic platforms—such as the da Vinci and KUKA systems—have demonstrated efficacy in pituitary-adenoma resection, sinonasal-tumor management, cochlear implantation, and mastoid drilling. The benefits reported include improved access, reduced instrument collision, tremor filtration, and enhanced surgical ergonomics. Institutional data and case studies indicate outcomes comparable or superior to those of the traditional approaches in select cases. Nonetheless, challenges remain, including high costs, limited training availability, and technical complexity.

**Conclusion:**

Robotic-assisted surgery has demonstrated clinical value in skull-base surgery, and it has the potential of improving outcomes while minimizing surgeon fatigue. Broader adoption will depend on cost reduction, widespread training, and technological advancements. Emerging innovations—including the integration of artificial intelligence, image-guided navigation, and instrument miniaturization—may further expand its usefulness in complex skull-base interventions.

## Introduction

The base of the skull is a highly-complex anatomical region located at the junction of the cranial cavity and facial structures. It supports critical neurovascular structures, including the brainstem, cranial nerves, and major blood vessels. Its intricate anatomy, combined with the need to preserve surrounding delicate structures, presents substantial challenges for surgeons performing traditional open or endoscopic procedures. Approaching the skull base requires passage through narrow and labyrinthine corridors, which increases the risk of complications such as nerve and vessel injuries. 1

Robotic-assisted surgery (RAS) has emerged as a transformative approach to minimally-invasive surgery. The enhanced visualization, precision, and dexterity provided by robotic systems have enabled access to anatomical regions that were previously difficult to reach. In otorhinolaryngology, the adoption of robotic platforms for procedures such as transoral robotic surgery (TORS) has paved the way for the exploration of their role in skull-base surgery. 2 3 Foundational work with platforms such as the da Vinci Surgical System (Intuitive Surgical, Inc.) and early institutional feasibility studies have demonstrated the potential of robotics to improve access and outcomes in anterior and lateral skull-base surgery. 4 5 6

Despite these developments, the published reviews have not comprehensively addressed the combined applications of RAS in anterior and lateral skull-base approaches, nor have they fully discussed emerging technologies and system-level challenges. The present review aims to provide a comprehensive exploration of the current applications, challenges, and future directions of RAS in skull-base procedures. The discussion covers anterior and lateral skull-base approaches in the field of otorhinolaryngology, with an emphasis on technological developments, contributions by pioneering institutions, and the capabilities of robotics in overcoming the drawbacks of conventional techniques. 3 The current review also highlights emerging trends in automation driven by artificial intelligence (AI), affordability innovations, and miniaturized systems that are reshaping the future landscape of robotic skull-base surgery.

## Methods

### Selection Criteria

The present review focuses on studies published between 2000 and 2024, reflecting the period in which robotic platforms became clinically relevant for skull-base surgery. Eligible studies included preclinical peer-reviewed research, feasibility reports, case series, clinical trials, systematic reviews, and meta-analyses. The selection criteria were chosen in such a way as to assure high levels of evidence related to RAS applications in skull-base procedures. To maintain relevance, the studies were required to specifically address anterior and lateral skull-base surgeries in otorhinolaryngology and related disciplines, such as neuro-otology and rhinology. The exclusion criteria were non-robotic studies, editorials, letters without data, and reports not involving skull-base surgery. Only publications in English were considered. The reference lists of the studies included were manually checked for additional eligible reports.

The literature searches were conducted on major academic databases, including PubMed, Scopus, and Web of Science, ensuring comprehensive coverage of relevant studies. The key search terms included combinations of: *robotic-assisted surgery* , *skull-base surgery* , *anterior skull base* , *lateral skull base* , *robotics in otology* , *transoral robotic surgery (TORS)* , and *endoscopic robotic surgery* . These terms facilitated the identification of studies highlighting clinical applications and preclinical developments in robotic systems. 4

## Study Selection

The screening process was performed manually by the authors in two stages: title/abstract review against eligibility criteria and full-text review of the studies meeting these criteria. Selection was conducted independently by two reviewers, with discrepancies resolved through consensus.

### Data Extraction and Synthesis

From each study included, we extracted data on anatomical target (anterior versus lateral skull base), robotic platform, study design, reported outcomes (such as access, precision, feasibility), and safety signals. The findings were integrated into a narrative synthesis, given the heterogeneity in study designs and outcomes.

### Quality Appraisal

A formal risk-of-bias tool was not applied due to the diversity of study designs. Instead, a qualitative appraisal was undertaken, emphasizing the clarity of the study design, the adequacy of the follow-up, the reporting of complications, and the reproducibility of the outcomes. This approach aligned with the assessment provided in the “Risk-of-Bias and Quality Assessment Summary” subsection of the present article.

### Scope Definition

The scope of the review is limited to RAS interventions targeting the anterior and lateral skull base. Specific emphasis is placed on applications within ear, nose, and throat (ENT) specialties, including rhinology for anterior skull-base tumors (such as sinonasal malignancies and pituitary adenomas) and otology for lateral skull-base conditions such as acoustic neuromas and cholesteatomas. This focus ensures that the review captures the nuances of robotic technology as applied to skull-base surgeries. 5

## Results

A targeted review of the literature yielded several studies on the application of RAS in anterior and lateral skull-base procedures. The selected studies included preclinical investigations, feasibility analyses, and early clinical applications reflecting the evolving nature of this surgical domain. Most studies focused on specific anatomical regions, including the pituitary fossa, sinonasal tract, mastoid, and cerebellopontine angle. Technologies such as the da Vinci Surgical System and KUKA platforms (KUKA AG) were the most frequently reported. Table 1 summarizes representative studies reviewed, 1 3 4 8 9 10 11 12 21 23 detailing their focus and main findings regarding surgical feasibility, precision, and innovation.

**Table 1:** Summary of key studies on robotic-assisted skull-base surgery

Study	Study focus/findings	Key outcomes
Matinfar et al. 1	Overview of robot-assisted skull-base surgery feasibility and system control	Established early feasibility of robot-assisted skull-base surgery
Blanco and Boahene 3	Preclinical study on robotic-assisted skull-base surgery	Highlighted training limitations and the need for specialized curricula
Kim and Zanation 4	Transoral robotic surgery (TORS) to resect skull-base tumors via transpalatal and lateral pharyngeal approaches	Improved access and surgeon ergonomics; effective tumor access
Chauvet et al. 8	First clinical study on TORS for sellar tumors	Feasibility confirmed; reduced morbidity reported
Troise et al. 9	Systematic review on TORS in maxillofacial surgery	Identified current capabilities and future potential of TORS
Garcia et al. 10	Initial experience with robotic-assisted otologic and lateral skull-base surgery	Reported enhanced accuracy and reduced iatrogenic injury
Weber et al. 11	Instrument flight to the inner ear with robotic precision	Demonstrated submillimetric accuracy in cochlear access
Federspil et al. 12	Experimental robotic milling in the calvarium and mastoid	Validated robotic milling for cavity formation with force control
Lee et al. 21	TORS for the skull base (cadaver study)	Showed feasibility for anterior skull-base access with reduced collateral damage
Ishida et al. 23	Situational-aware force control in robotic drilling	Improved safety and precision for robotic skull-base procedures

## Anterior Skull Base – Reported Approaches

Robotic systems were used in transnasal and transoral corridors to access anterior skull-base lesions. These include sinonasal malignancies and pituitary adenomas. 6 7 Transoral robotic surgery was also employed for anterior skull base access, with documented feasibility in selected studies. 8 9

## Lateral Skull Base – Reported Applications

In lateral skull-base procedures, robotic technology was applied to otologic surgeries such as mastoid drilling, cochlear implantation, and approaches to acoustic neuromas. 10 11 Robotic-milling techniques were demonstrated in experimental models for cavity creation in the calvarium and mastoid. 12

## Institutional Contributions

Several academic institutions have contributed to the development and application of robotic systems in skull-base surgery. A summary of key institutions, focus areas, and contributions is provided in Table 2 .

**Table 2:** Institutional contributions to robotic-assisted skull-base surgery

Country	Institution	Focus Area	Contribution
United States	Johns Hopkins Medicine 13	Anterior skull-base surgeries	Application of Transoral robotic surgery (TORS) for skull-base access
United States	Mayo Clinic 14	Lateral skull-base surgeries	Development of robotic-assisted otologic techniques
United Kingdom	University College London 16 (UCL)	Endoscopic, endonasal skull-base surgery	Robotic-handle prototypes to enhance dexterity
Germany	Saarland University Hospital 12	Lateral skull-base surgeries	Precision in robotic milling applications
France	Zimmer Biomet Robotics 18	Neurosurgical skull-base procedures	Integration of frameless stereotactic navigation
Japan	Fujita Health University 19	Transoral approaches to the skull base	Feasibility studies using the da Vinci Surgical System for upper cervical access 19

### United States

In the United States, several institutions have pioneered skull-base RAS. Leading centers such as Johns Hopkins Medicine 13 and the Mayo Clinic 14 have conducted significant research and clinical trials on the application of robotic systems for anterior and lateral skull-base procedures. The adoption of the da Vinci Surgical System has played a critical role in these advancements, enabling surgeons to navigate complex anatomy with precision. 15

At Johns Hopkins Medicine, 13 researchers have focused on improving TORS techniques to access the anterior skull base with minimal morbidity. Similarly, the Mayo Clinic 14 has contributed to the development of robotic systems for lateral skull-base procedures, emphasizing innovations in otologic surgeries for conditions such as cholesteatomas and acoustic neuromas.

### United Kingdom

At University College London (UCL), researchers have developed robotic handle prototypes aimed at enhancing dexterity and ergonomics in endoscopic endonasal skull-base surgeries. A preclinical randomized controlled trial 16 demonstrated that these robotic handles significantly improved surgical performance and reduced surgeon fatigue compared to conventional instruments. This work underscores the United Kingdom's commitment to developing innovative tools for minimally-invasive procedures.

### Germany

German centres have contributed to the development of robotic technologies for skull-base surgery. Work at Saarland University Hospital has explored robotic approaches to the lateral skull base aimed at improving surgical access and precision within anatomically complex regions. In addition, German research groups have pioneered robotic milling systems capable of achieving sub-millimetric accuracy in skull-base procedures, supporting safer and more precise bone removal in delicate areas. 12 Robotic platforms have also been adapted for extended endoscope-assisted transsphenoidal surgery, demonstrating the feasibility of integrating hexapod-based robotic systems into minimally invasive skull-base techniques. 17

### France

The ROSA ONE Brain robotic system, 18 developed by Zimmer Biomet Robotics (formerly Medtech) in France, has become a key tool in neurosurgical procedures, including skull-base surgery. This system integrates neurosurgical planning software and a high-precision robotic arm, enabling frameless stereotactic procedures and enhanced surgical accuracy. The ROSA ONE Brain system has been instrumental in expanding the capabilities of minimally-invasive neurosurgery across Europe.

### Japan

At Fujita Health University, researchers 19 have explored the feasibility of transoral robotic-assisted neurosurgery using the da Vinci Surgical System. They have focused on accessing the skull base and upper cervical spine, emphasizing suturing techniques and the reach of the transoral approach. This work has significantly advanced the application of robotics in neurosurgery and deep-seated lesion management.

### South Korea and China

In addition to Japan, countries such as South Korea and China are actively exploring robotic applications in skull-base surgery. These efforts include integrating artificial intelligence into robotic systems and developing cost-effective platforms to improve accessibility in complex surgical interventions.

## Discussion

Robotic-assisted surgery in skull-base procedures remains an evolving field characterized by technical promise and constrained clinical adoption. A focused analysis of key published studies reveals concentrated efforts regarding anterior and lateral skull-base interventions, with varying levels of clinical maturity and methodological robustness.

Robotic-assisted surgery has shown great promise in the treatment of anterior skull-base lesions, including sinonasal tumors and pituitary adenomas. The traditional endoscopic transnasal approaches to these conditions have been aided by the enhanced precision and visualization provided by robotic systems. Systems such as the da Vinci Surgical System enable surgeons to manipulate tissue with greater dexterity in narrow surgical corridors, with three-dimensional (3D) visualization and dampening of hand tremors. 6 This makes it particularly advantageous for navigating the complex anatomy of the anterior skull base.

Robotic systems also facilitate improved resection margins in sinonasal tumors and pituitary adenomas, reducing the risk of complications. Robotic assistance has demonstrated 7 its usefulness in minimizing collateral damage to critical structures, such as the optic chiasm and carotid arteries, for example, while providing access to the sphenoid and sellar regions.

Several case studies exemplify the success of robotic interventions in anterior skull-base surgeries. Researchers 8 9 have demonstrated the feasibility of robotic-assisted approaches for pituitary adenomas using the TORS technique, and they have reported reduced operative times, improved tumor resection, and fewer postoperative complications compared to conventional methods. Such evidence underscores the transformative role of robotic systems in anterior skull-base procedures.

In lateral skull-base surgery, robotic technology has advanced surgical precision for conditions such as acoustic neuromas and cholesteatomas. 10 These procedures require careful preservation of critical structures such as the facial nerve and cochlear apparatus. The KUKA robotic system has been applied in mastoid drilling and cochlear implantation with enhanced accuracy and reduced iatrogenic injury. 11

Robotic milling techniques have been explored in experimental models. Federspil et al. 12 demonstrated the feasibility of cavity formation with controlled force in the calvarium and mastoid. Such techniques may lead to more reproducible outcomes in future skull-base surgery.

Robotic systems also enable the simultaneous use of endoscopes and instruments in narrow anatomical corridors, which is relevant to optimize visualization and improve access, particularly in lateral skull-base surgery. 10 11 12 These advantages support the expanding role of robotic platforms in minimizing surgical trauma and enhancing patient outcomes.

### Benefits of Robotic Surgery in Skull-Base Procedures

Robotic-assisted surgery has revolutionized skull-base procedures by providing unparalleled precision and visualization in narrow anatomical spaces. Most of the traditional approaches have a limitation of restricted access and poor visibility, thus increasing the chances of iatrogenic injury. High-definition 3D visualization and advanced maneuverability of robotic instruments, such as those from the da Vinci Surgical System, overcome the drawbacks of these challenges. This has resulted in better resection margins, fewer cases of surgical trauma, and an overall improved outcome for the patients. 20 Lee et al. 21 demonstrated the effectiveness of robotic systems in achieving precise tumor resection with minimal collateral damage in anterior skull-base surgeries, such as those targeting pituitary adenomas and sinonasal malignancies. Beyond patient outcomes, robotic systems also significantly enhance the surgical experience by reducing the physical strain on surgeons. 15 16 Traditional open and endoscopic skull-base surgeries require awkward body positioning, leading to fatigue and musculoskeletal strain. Robotic systems enable surgeons to operate from a seated console with ergonomic controls, mitigating these physical demands. Kim and Zanation 4 noted that robotic assistance improves surgeon comfort during long procedures, reducing fatigue and improving precision.

Robotic systems also facilitate tremor elimination and enhance dexterity, enabling the precise manipulation of instruments in confined surgical fields. 22 These benefits are particularly crucial for complex skull-base procedures, in which minute errors can have serious consequences.

### Challenges and Limitations

The high cost of robotic systems is one of the most significant barriers to their widespread adoption in skull-base surgery. Systems such as the da Vinci Surgical System involve substantial initial investment, along with ongoing maintenance and training expenses. These costs are prohibitive for many hospitals, particularly in low- and middle-income countries. The high cost of the adoption of robotic systems means that, even in high-income regions, availability and accessibility are limited by their financial burden.

Additionally, the cost of consumables, such as robotic instruments and disposable components, adds to the economic challenges. In turn, such economic strain might be shifted toward patients and restrict access for people who cannot afford it. 1

Another major limitation is the steep learning curve associated with skull-base RAS. While robotic systems offer advanced capabilities, their effective use requires specialized training and experience. Surgeons must become proficient not only in operating the robotic system but also in adapting traditional surgical techniques to robotic platforms. Blanco and Boahene 3 highlighted the limited availability of training programs tailored specifically to skull-base surgery, which hinders the development of a skilled workforce.

Furthermore, the lack of standardization in training curriculums in skull-base RAS results in a variability in surgeons' skills and knowledge. This inconsistency may contribute to data bias in surgical outcomes across institutions. Consequently, achieving uniform or comparable results in skull-base RAS remains a challenge.

### Risk-of-Bias and Quality Assessment Summary

The current narrative review includes data drawn from a heterogeneous body of literature comprising preclinical studies, case series, feasibility reports, and systematic reviews. Given the diversity of study designs, a formal risk-of-bias assessment was not uniformly applicable. However, a qualitative appraisal of methodological rigor was performed to identify potential sources of bias and limitations in the evidence base.

Several of the studies included were preclinical in nature, involving cadaveric models or experimental systems without clinical validation (such as those by Ishida et al. 23 and Federspil et al. 12 ), which inherently limits generalizability to the operative practice. Clinical studies such as those by Chauvet et al. 8 and Garcia et al. 10 provided valuable insights into feasibility and early outcomes, but they were often limited by small sample sizes, absence of control groups, and short follow-ups. These factors contribute to a moderate-to-high risk of selection and publication bias.

Additionally, the reporting standards varied considerably across studies, with inconsistent documentation of complication rates, learning curves, and long-term patient outcomes. The absence of randomized controlled trials in this domain further limits the strength of the inferences that can be drawn regarding clinical efficacy and safety.

Overall, while the reviewed literature supports the potential usefulness of skull-base RAS, the evidence remains preliminary in nature. High-quality prospective studies with standardized protocols, adequate power, and long-term follow-up are required to establish definitive clinical benefits and cost-effectiveness.

### Future Directions and Promises

The future of skull-base RAS lies in the continuous evolution of robotic technologies, particularly the integration of AI, which has the potential of enhancing surgical precision by providing real-time intraoperative decision-making support and advanced image-guided navigation. This includes features such as predictive modeling of surgical outcomes, automated identification of critical structures, and adaptive instrument control. Ishida et al. 23 highlighted the development of situational-aware force control mechanisms in robotic systems, which enable safer interactions with delicate anatomical structures during skull-base drilling.

Another promising avenue is the miniaturization of robotic instruments. Future robotic systems are likely to be equipped with smaller, more flexible tools that will more easily access challenging anatomical regions. Miniaturization efforts have resulted in the creation of flexible, tendon-driven robotic systems with diameters as short as 3 mm, enhancing maneuverability in confined anatomical corridors. Researchers 24 at the University of California, Riverside, for example, have developed a telescopic tendon-driven needle robot designed for minimally-invasive neurosurgery, demonstrating precise navigation capabilities within the brain. Furthermore, the development of handheld robotic devices with detachable end-effectors offers improved dexterity and ergonomics in endoscopic, endonasal skull-base surgeries. These systems comprise interchangeable articulated instruments that expand the operative workspace and enhance the surgeon's dexterity. 25

Addressing the high costs of robotic systems is crucial to increase their accessibility. Strategies such as the development of cost-effective robotic platforms and modular systems could reduce the financial burden on healthcare institutions. Collaboration between manufacturers and academic institutions could drive innovation in low-cost robotics tailored for specific surgical applications. Federspil et al. 12 emphasized that achieving cost efficiency is critical to expand the use of robotics in lateral skull-base surgery and beyond.

Expanding training programs is equally important. Standardized curricula and simulation-based training modules could shorten the learning curve and produce more skilled robotic surgeons. Additionally, interdisciplinary collaborations among neurosurgeons, otolaryngologists, and engineers can foster innovation and facilitate the adoption of robotics across diverse surgical specialties.

## Conclusion

Robotic-assisted surgery is promising to change the face of skull-base surgery by enhancing precision, visualization, and ergonomics, thereby addressing many limitations of the traditional approaches. Evidence from anterior and lateral procedures highlights its potential to improve patient outcomes in complex anatomical regions.

Despite these advantages, widespread adoption remains hindered, because of their high costs and steep learning curves. Advances in technology, particularly AI integration, miniaturization of instruments, and cost-effective platforms, may progressively lower these barriers and expand clinical accessibility.

Future priorities include fostering multidisciplinary collaboration, expanding structured training programs, and undertaking large, prospective clinical series to clarify the role of robotics in skull-base surgery. As these developments mature, skull-base RAS is likely to become an established component of the minimally-invasive practice.
